# Inhaler Technique Questionnaire (InTeQ) in pediatric patients with asthma

**DOI:** 10.1007/s12519-023-00695-w

**Published:** 2023-03-06

**Authors:** Catalina Lizano-Barrantes, Olatz Garin, Alexandra Lelia Dima, Karina Mayoral, Angels Pont, Eva María Ortiz, María Araceli Caballero-Rabasco, Manuel Praena-Crespo, Laura Valdesoiro-Navarrete, María Teresa Guerra, Alberto Bercedo-Sanz, Gimena Hernández, Camila Maroni, Inés de Mir, María Ángeles Carrasco, Marta Ortega, Alberto Servan, José Antonio Castillo, Eva Tato, Montse Ferrer, the ARCA Group, Yolanda Pardo, Víctor Zamora, Isabel Moneo, Olga Cortés, Eric van Ganse, Marijn de Bruin

**Affiliations:** 1 Health Services Research Group, IMIM (Hospital del Mar Medical Research Institute), Barcelona Biomedical Research Park, Doctor Aiguader, 88, 08003 Barcelona, Spain; 2 Department of Medicine and Life Sciences, Universitat Pompeu Fabra, Barcelona, Spain; 3 Department of Pharmaceutical Care and Clinical Pharmacy, Faculty of Pharmacy, Universidad de Costa Rica, San Jose, Costa Rica; 4 Centro de Investigación Biomédica en Red de Epidemiología y Salud Pública CIBERESP, Madrid, Spain; 5 Research and Development Unit, Institut de Recerca Sant Joan de Déu, Barcelona, Spain; 6 Department of Pediatrics, Obstetrics and Gynaecology and Preventive Medicine, Universitat Autònoma de Barcelona, Barcelona, Spain; 7 Pediatric Allergy and Pulmonology Unit, Pediatric Service, Hospital del Mar, Barcelona, Spain; 8 Pediatric Allergy and Pulmonology Unit, Pediatric Service, Hospital Universitari Parc Taulí, Sabadell, Spain; 9 Centro de Salud La Candelaria, Servicio Andaluz de Salud, Seville, Spain; 10 Grupo de Vías Respiratorias de La Asociación Española de Pediatras de Atención Primaria (AEPAP), Madrid, Spain; 11 Centro de Salud Jerez Sur, Servicio Andaluz de Salud, Seville, Spain; 12 Centro de Salud La Sagrera, Barcelona, Spain; 13 Centro de Salud Los Castros, Cantabria, Spain; 14 Facultat de Medicina, Universitat Autònoma de Barcelona, Barcelona, Spain; 15 Pediatric Allergy and Pulmonology, and Cystic Fibrosis Unit, Hospital Universitari Vall d’Hebron, Barcelona, Spain; 16 Consultorio Sevilla la Nueva, Madrid, Spain; 17 Centro de Salud Dos de Mayo, Madrid, Spain; 18 Hospital Miguel Servet, Zaragoza, Spain; 19 Hospital Universitario Araba, Vitoria‑Gasteiz, Spain; 20 Departamento de Farmacologia, Pediatría y Radiología, Universidad de Sevilla, Seville, Spain; 21 Institut d’Investigació i Innovació Parc Taulí, Sabadell, Spain

Asthma in school-aged children is a major public health problem worldwide [1, 2]. Inhaled medications are the mainstay of its pharmacological management [2], but only 8%–22% of children with asthma use their inhalers correctly [3]. Asthma clinical outcomes are poor in children [4], largely due to inhaler technique [5, 6].

Since inhalation technique is a key modifiable factor for treatment success, regular monitoring is essential [2]. Using inhaler devices correctly can be difficult [7] and the technique deteriorates over time [8]. However, inhalation technique assessment is not common in real life [9], which could lead to an unjustified treatment escalation. Consequently, identifying feasible and valid methods to assess it is of great importance [10]. Questionnaires or checklists to measure the inhaler technique remain the easiest, most accessible, and most commonly used method [2, 5].

Systematic reviews [5, 10, 11] highlight the considerable variation among the inhaler technique checklists used by healthcare professionals. Specifically, the sources used to develop the content [10] (manufacturers’ leaflets, guidelines, previous studies), the distinction between critical and non-critical steps [5, 10], the number of inhalation technique steps (from 3 to 21), or evidence of their validity and reliability [10].

Several studies have used a general evaluation of patients’ confidence in their inhaler technique [12, 13], while we have found only three step-by-step patient-reported questionnaires [14–16], all of which were validated in adults. Two are specifically for metered-dose inhalers (MDI), with nine [14] and twenty [15] items, and the most recent one, the Inhaler Technique Questionnaire (InTeQ) [16], includes five items common to MDI and dry powder inhalers (DPI).

The InTeQ has proven to be feasible, valid, and reliable in adults with persistent asthma [16], which can be useful for patients’ self-monitoring and healthcare professionals teaching patients. This study aimed to assess the InTeQ’s validity and reliability in children and adolescents with asthma. This study was performed within the ARCA (Asthma Research in Children and Adolescents) cohort, a prospective, multicenter, observational study (NCT04480242) [17], replicating the original InTeQ validation performed on adults with asthma [16].

Patients were recruited in five outpatient pediatric pulmonology hospital units and nine primary care pediatric centers in Spain (2018–2022), with the following inclusion criteria: age 6–14 years, clinical diagnosis of asthma, treatment with inhaled corticosteroids (alone or combined with long-acting beta-agonists) for more than six months in the previous year, and access to a smartphone. Exclusion criteria were other respiratory diseases. Written informed consent was requested for all participants.

The InTeQ was collected through computer-assisted telephone interviews (CATIs), together with a question on spacer use, details on asthma treatments, and the Asthma Control Questionnaire (ACQ-symptoms) [18]. Two versions of the CATIs were administered according to age: proxy or self-response.

InTeQ items ask the frequency of performing five key steps when using the inhaler in the previous six months with a five-level Likert scale (from “Always” to “Never”) [16]. A global score was calculated as a sum of the items answered "Always," categorized into Good, Fair, or Poor inhaler technique.

The InTeQ psychometric properties were assessed with data from CATIs at baseline. We examined the InTeQ’s structural validity using Mokken scaling analysis. Our sample (319 participants) was above the minimum size of 250 estimated sufficient to establish scalability when only one cluster of items was identified as a Mokken scale and the strength was moderate [19]. Reliability was estimated with Cronbach’s alpha coefficient, based on the internal consistency among items.

We evaluated construct validity by assessing the ability of the InTeQ to discriminate among known groups defined by the ACQ and the use of spacer. On one hand, the hypothesis raised a priori that patients with well-controlled asthma have better inhaler technique [5, 6]. On the other hand, based on the recommendation of the Aerosol Drug Management Improvement Team (ADMIT) [20] of substituting three steps with the “tidal breathing” maneuver (inhale and exhale five times slowly) for spacers, we expected children using them to perform less frequently the following InTeQ items: “Breathe out fully before”, “Breathe in deeply”, and “Hold breath for at least 10 seconds”.

Moreover, we conducted a face-to-face inhalation technique assessment in a subsample of 37 participants during a visit to their healthcare center. Participants were asked to bring their inhaler device and spacer if applicable, and instructed to “perform their inhalation as usual”. Two independent experts, a pediatrician and a member of the research team, observed and rated each step as: ++ (correctly performed), + (poorly performed), or − (not performed). Patients either answered the InTeQ first and then were observed, or vice versa, to adjust for any potential bias associated with the order of data collection. The experts were blinded to results from the patient-reported technique.

We evaluated criterion validity by estimating the agreement between the patient-reported inhalation technique through InTeQ and the experts’ observation (gold standard) in the subsample (size calculated to estimate an overall agreement percentage of 70% with a precision of ± 15%). Crude agreement and Kappa coefficients were calculated between pediatrician observation and InTeQ responses and between the two experts' observations.

Data were analyzed using R (version 4.2.0), RStudio (version 1.1.463), and the Mokken package in *R*.

Of the 323 participants answering the baseline interview, 319 (98.7%) completed the InTeQ. Supplementary Table 1 shows that most patients were treated with inhaled corticosteroids combined with long-acting β-agonists in a fixed dose (68.0%), with MDI (77.4%), and reported always using a spacer during the last six months (85.3%). The face-to-face assessment subsample showed similar characteristics.

Figure 1 shows that the frequency of InTeQ items responded with “Always” varied from 43% to 90%. The second most frequent response was “Never” (27%–33%), except for “Breathe in deeply” (“Sometimes” 9%). None of the participants answered “Don’t know.”

**Fig. 1:**
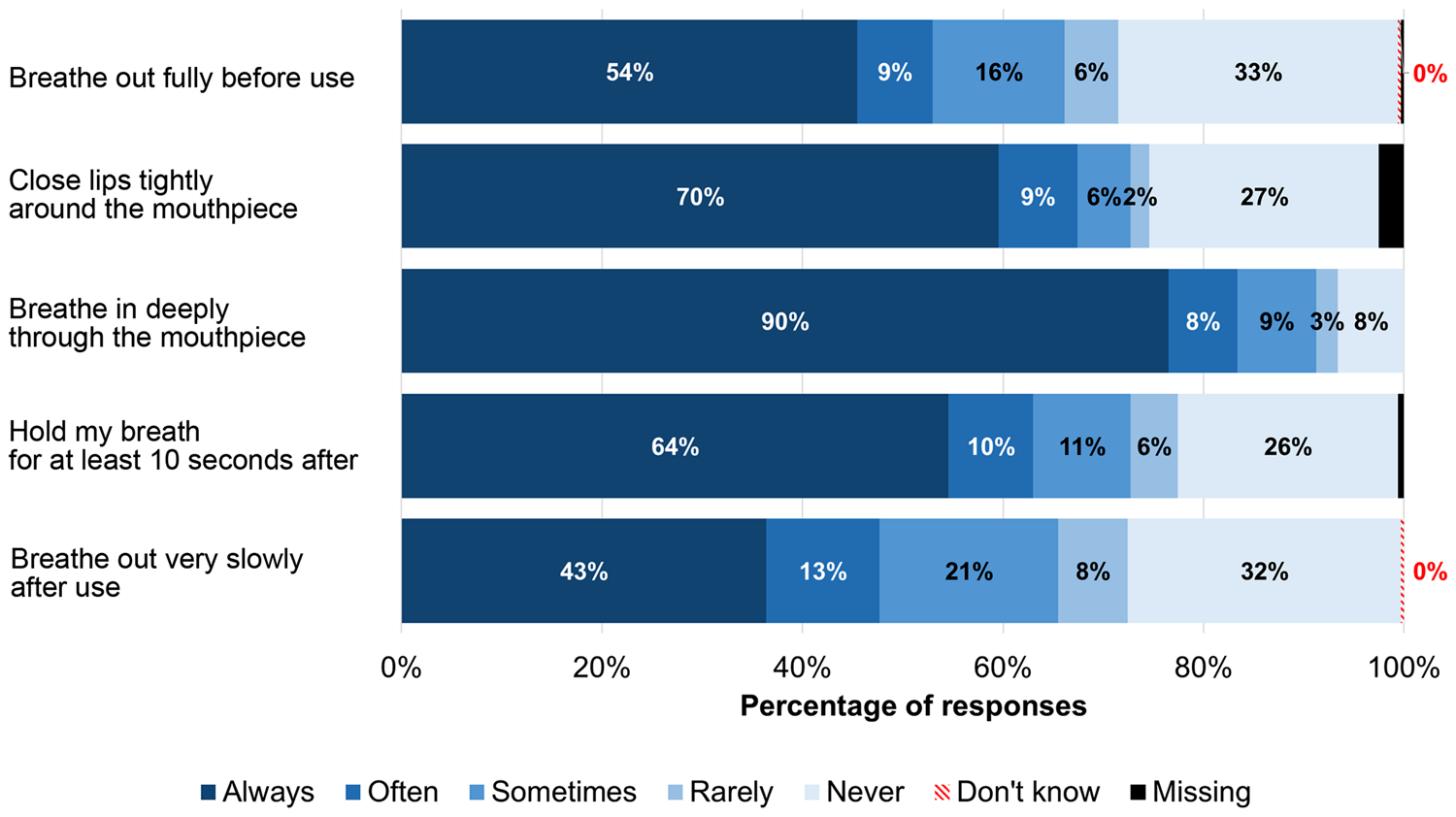
Distribution of InTeQ items (frequency of performing the step during the last 6 months)

Supplementary Table 2 shows that InTeQ items present a skewed distribution and their coefficients of homogeneity are above the cut-off point of 0.3, except for the item ‘Close lips tightly.’ An automated item selection procedure indicated that, at homogeneity threshold levels of 0.30–0.35, the remaining four InTeQ items could form a single scale. Cronbach’s alpha coefficient was 0.613 and 0.655 for the InTeQ with five and four items, respectively.

Table 1 shows construct validity results, with statistically significant differences according to asthma control groups in the item ‘Close lips tightly’ and the InTeQ global score with five items (*P* = 0.006 and 0.025, respectively). Furthermore, significant differences between spacer users and non-users were found in two of the three expected items (“Breathe out fully before” and “Hold breath after”), and in InTeQ global scores calculated both with its five items (*P* = 0.021) and with only four items (*P* = 0.005).

**Table 1:** Validity of InTeQ item and global scores, comparing known groups defined by asthma control and use of spacer

Items	ACQ		Use of spacer	
	Well-controlled (*n* = 202)	Intermediate—not well-controlled (*n* = 117)	Non-users (*n* = 65)	Users (*n* = 240)
*Breathe out fully before*				
Always	94 (46.5%)	51 (43.6%)	43 (66.2%)	97 (40.8%)
Often–sometimes	39 (19.3%)	27 (23.1%)	14 (21.5%)	50 (21.0%)
Rarely–never	67 (33.2%)	39 (33.3%)	8 (12.3%)	91 (38.2%)
Don't know	1 (0.5%)	0 (0.0%)	0 (0.0%)	1 (0.4%)
Missing	1 (0.5%)	0 (0.0%)	0 (0.0%)	1 (0.4%)
* P* value	0.725		< 0.001*	
*Close lips tightly*				
Always	133 (65.8%)	57 (48.7%)	43 (66.2%)	138 (59.5%)
Often–sometimes	23 (11.4%)	19 (16.2%)	12 (18.5%)	28 (12.1%)
Rarely–never	40 (19.8%)	39 (33.3%)	10 (15.4%)	66 (28.4%)
Don't know				
Missing	6 (3.0%)	2 (1.7%)	0 (0.0%)	8 (3.3%)
* P* value	0.006*		0.071	
*Breathe in deeply*				
Always	161 (79.7%)	83 (70.9%)	52 (80.0%)	180 (75.0%)
Often–sometimes	26 (12.9%)	21 (17.9%)	7 (10.8%)	38 (15.8%)
Rarely–never	15 (7.4%)	13 (11.1%)	6 (9.2%)	22 (9.2%)
Don't know				
Missing				
* P* value	0.203		0.589	
*Hold breath after*				
Always	114 (56.4%)	60 (51.3%)	45 (69.2%)	124 (52.1%)
Often–sometimes	33 (16.3%)	25 (21.4%)	12 (18.5%)	42 (17.6%)
Rarely–never	53 (26.2%)	32 (27.4%)	8 (12.3%)	72 (30.3%)
Don't know				
Missing	2 (1.0%)	0 (0.0%)	0 (0.0%)	2 (0.8%)
* P* value	0.493		0.011*	
*Breathe out slowly after*				
Always	75 (37.1%)	41 (35.0%)	29 (44.6%)	84 (35.1%)
Often–sometimes	55 (27.2%)	38 (32.5%)	21 (32.3%)	66 (27.6%)
Rarely–never	71 (35.1%)	38 (32.5%)	15 (23.1%)	89 (37.2%)
Don't know	1 (0.5%)	0 (0.0%)	0 (0.0%)	1 (0.4%)
Missing				
* P* value	0.625		0.099	
Quality of inhalation technique according to InTeQ global score (5 items)				
Poor (0–2 "Always")	74 (36.6%)	61 (52.1%)	18 (27.7%)	109 (45.4%)
Fair (3 "Always")	55 (27.2%)	23 (19.7%)	17 (26.2%)	58 (24.2%)
Good (4–5 "Always")	73 (36.1%)	33 (28.2%)	30 (46.2%)	73 (30.4%)
* P* value	0.025*		0.021*	
Quality of inhalation technique according to InTeQ global score (4 items)				
Poor (0–1 "Always")	60 (29.7%)	46 (39.3%)	10 (15.4%)	88 (36.7%)
Fair (2 "Always")	53 (26.2%)	27 (23.1%)	20 (30.8%)	59 (24.6%)
Good (3–4 "Always")	89 (44.1%)	44 (37.6%)	35 (53.8%)	93 (38.8%)
* P* value	0.213		0.005^†^_*_	

ACQ: Asthma Control Questionnaire, which assesses the frequency of 5 asthma symptoms during the previous week on a 7-level Likert scale from 0 (no impairment) to 6 (maximum impairment). The overall score, calculated as the mean item responses, ranges from 0 to 6. Cut-off points of 1.5 and 0.75 define not well- and well-controlled asthma, respectively [18]. Differences were tested using Chi-square, *P < 0.05

The results of the 37 participants’ face-to-face assessment are summarized in Fig. 2. Their treatment was administered with MDI in 32 (26 with a spacer) and with DPI in 5. Most participants responded “Always” to all items of the InTeQ (36.1%-67.6%). According to the pediatricians’ observation, none of the 37 participants performed all the steps correctly. Most of them (81.1%) skipped the first step (“Breathe out fully before”) completely. However, most participants (78.4%) performed the step ‘Close lips tightly’ correctly. Supplementary Table 3 shows that the percentage of agreement between the observation by pediatricians and researchers ranged from 77.8 to 100%, and kappa coefficients were from substantial (0.642) to perfect (1.00).

**Fig. 2:**
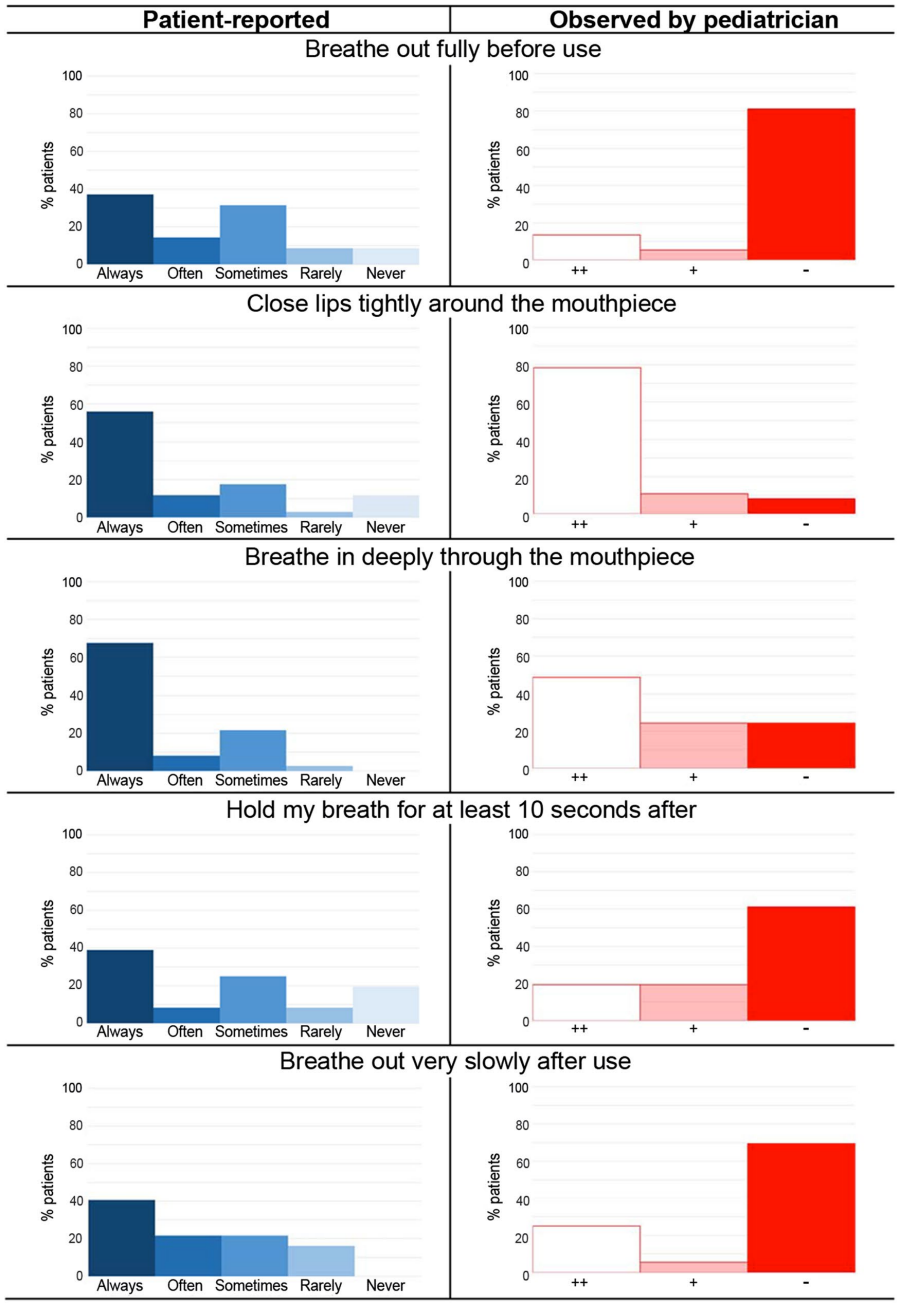
Inhaler technique reported by patients with the InTeQ and assessed by pediatrician through face-to-face observation

The highest agreement between the participants’ reported inhalation technique through the InTeQ and the pediatricians’ observation (Table 2) was obtained in items “Close lips tightly” (87.9%) and “Breathe in deeply” (77.8%), the rest presenting percentages of agreement lower than 40%. Kappa coefficients ranged from poor to fair (−0.112 to 0.298).

**Table 2:** Agreement between patient-reported inhalation technique (InTeQ) and pediatricians’ observation

Items	Observation		% agreement (95%CI)	Kappa (SE)
	++/+	−		
*Breathe out fully before*				
Always to sometimes	7 (20.0%)	22 (62.9%)	37.1 (21.1–53.2)	0.098 (0.051)
Rarely or never	0 (0.0%)	6 (17.1%)		
*Close lips tightly*				
Always to sometimes	28 (84.8%)	0 (0.0%)	87.9 (76.7–99.0)	0.298 (0.234)
Rarely or never	4 (12.1%)	1 (3.0%)		
*Breathe in deeply*				
Always to sometimes	27 (75.0%)	8 (22.2%)	77.8 (64.2–91.4)	0.158 (0.142)
Rarely or never	0 (0.0%)	1 (2.8%)		
*Hold breath after*				
Always to sometimes	9 (25.0%)	17 (47.2%)	38.9 (23.0–54.8)	− 0.112 (0.137)
Rarely or never	5 (13.9%)	5 (13.9%)		
*Breathe out slowly after*				
Always to sometimes	9 (25.0%)	22 (61.1%)	33.3 (17.9–48.7)	− 0.041 (0.089)
Rarely or never	2 (5.6%)	3 (8.3%)		

The expert observation was dichotomized into correctly/poorly performed (++/+) and not performed (−). The InTeQ responses of frequency were dichotomized into “Always-Often-Sometimes” or “Rarely–Never.” Kappa coefficient values: 0.0–0.2 (slight agreement), 0.2–0.4 (fair), 0.4–0.6 (moderate), 0.6–0.8 (substantial), and 0.8–1.0 (almost perfect). *CI* confidence interval, *SE* standard error

This is the first study describing the validation of an instrument for assessing inhaler technique with any type of device in pediatric patients with asthma. The high response rate and low proportion of missing values suggest an easy completion. The InTeQ showed good feasibility, evidence of unidimensionality with four items, acceptable reliability, and good construct validity. However, the agreement between the patient-reported inhalation technique and expert observation was poor, as most of the participants answered “Always” to all InTeQ items, but none of them performed all the steps correctly according to the experts.

In a systematic review [11] which evaluated the errors in inhalation technique based also on five steps common in MDIs and DPIs, only one of the five steps considered, “prepare the device (uncap)”, differs from InTeQ, which included “Breathe out slowly after” instead. Furthermore, “not removing the cap” along with “not having the head tilted (chin slightly upward)” have been associated with uncontrolled asthma [21]. Hence, it would be worth considering the advantage of adding a step to the InTeQ without increasing the burden unnecessarily.

When examining the InTeQ’s structural validity, the lower homogeneity of item “Close lips tightly” could be explained by the high use of spacers in children and adolescents (85.3% in our sample). However, since it is also required when using a spacer [2, 20], we would advise maintaining the original version of the questionnaire with five items, and to calculate the global score only with the four InTeQ items that demonstrated unidimensionality in children.

The InTeQ was able to discriminate among known groups consistently with hypothese, indicating the adequate construct validity of the questionnaire. These findings are consistent with previous studies that identified better inhalation technique associated with better asthma control [5, 6, 21]. The item “Close lips tightly” presented statistically significant differences per asthma control (*P* = 0.006), consistent with a previous study [21] that found that the lack of lip sealing around the mouthpiece was associated with a higher rate of exacerbations in adult patients with asthma. Therefore, these findings also support maintaining the item “Close lips tightly” in the InTeQ.

Current guidelines [2, 20] recommend the steps included in the InTeQ for pediatric patients. However, the ADMIT [20] proposes “tidal breathing” as the standard maneuver when using a spacer, which substitutes three of the five InTeQ steps. In our study, only around half the children using a spacer reported performing always two of the InTeQ steps replaceable with this maneuver (“Breathe out fully before” and “Hold breath for at least 10 seconds”), far from those not using a spacer (66% and 62%). In contrast, 75% of children using a spacer reported performing always the third replaceable step, ‘Breathe in deeply through the mouthpiece,’ similarly to children not using a spacer (80%), which could reflect a mixture of both types of inhaler technique.

The poor agreement between the patient-reported inhalation technique and observation was similar to findings from other studies [12–14]. The higher agreement when the patients performed the step and the lower when they were not performing it [14], also observed in our study, suggests that patients are frequently not aware when they are not performing a step. This finding supports the importance of asking for frequency of performance step-by-step instead of the patients' global confidence in their technique [13] or just assessing their theoretical knowledge [14, 15].

Some limitations of this study should be considered. First, the assessment of inhaler technique by expert observation is subjective, but the high interrater reliability obtained supports the suitability of expert observation as the gold standard. However, observation could impact on how patients use their inhalers, as this is not their usual situation. Second, the results on the agreement between observation and self-reporting should be interpreted with caution, considering the low number of participants.

Our findings suggest that the InTeQ is a feasible, reliable, and valid instrument for assessing inhalation technique in children and adolescents with persistent asthma. Its low administration burden facilitates its applicability in research and especially in clinical settings, where a frequent assessment of inhaler technique is advised. Due to the high proportion of poorly performed steps that patients were not aware of, it would be advisable for health professionals to combine observation with self-reporting of the inhaler technique to identify the aspects to improve. Finally, the need to develop a version of the InTeQ specially designed for spacer users merits further consideration.

## Supplementary Information

Below is the link to the electronic supplementary material.

Supplementary file1 (DOCX 31 KB)

## Data Availability

Data presented in this study are available upon reasonable request to the corresponding authors.
