# Supplementary material for: Inhaler Technique Questionnaire (InTeQ) in pediatric patients with asthma
Source: World J Pediatr. 2023 Mar 6;19(8):798–804. doi: 10.1007/s12519-023-00695-w (PMC10348940; doi:10.1007/s12519-023-00695-w)
Supplement: Supplementary file 1 — Supplementary file1 (DOCX 31 KB) [file 12519_2023_695_MOESM1_ESM.docx]

**Supplementary Table 1.** Patients’ characteristics at baseline in the ARCA sample and in the face-to-face assessment subsample

|  | **All patients**  (n=319) | **Subsample**  (n = 37) | ***p*-value** |
| --- | --- | --- | --- |
| Age, mean (SD) | 10.2 (2.5) | 9.6 (1.9) | 0.053 |
| Sex |  |  |  |
| Male | 176 (55.2%) | 15 (40.5%) | 0.052 |
| Female | 143 (44.8%) | 22 (59.5%) |  |
| Inhaled controller treatment |  |  |  |
| Corticosteroids | 96 (30.1%) | 12 (32.4%) | 0.832 |
| Fixed dose corticosteroids and LABA | 217 (68.0%) | 25 (67.6%) |  |
| Other medication | 5 (1.6%) | 0 (0.0%) |  |
| No medication | 1 (0.3%) | 0 (0.0%) |  |
| Inhaler type for controller treatment |  |  |  |
| Metered-Dose Inhaler (MDI) | 247 (77.4%) | 35 (94.6) | 0.024** |
| Dry-Powder Inhaler (DPI) | 58 (18.2%) | 2 (5.4%) |  |
| *Unknown* | *14 (4.9%)* | *0 (0.0%)* |  |
| Frequency of spacer use during the last 6 months |  |  |  |
| Always | 272 (85.3%) | 36 (97.3%) | 0.185 |
| Often | 0 (0.0%) | 0 (0.0%) |  |
| Sometimes | 8 (2.5%) | 0 (0.0%) |  |
| Rarely | 2 (0.6%) | 0 (0.0%) |  |
| Never | 37 (11.6%) | 1 (2.7%) |  |
| Frequency of reliever treatment use during the last 4 weeks |  |  |  |
| Every day | 15 (4.7%) | 3 (8.6%) | 0.009** |
| Almost every day | 27 (8.5%) | 6 (17.1%) |  |
| Once or twice every week | 81 (25.6%) | 14 (40.0%) |  |
| Less than once a week | 176 (55.5%) | 12 (34.3%) |  |
| Never | 18 (5.7%) | 0 (0.0%) |  |
| *Missing* | *2* | *2* |  |
| Asthma Control Questionnaire (ACQ) | 0.79 (1.0) | 1.1 (1.3) | 0.062 |
| Well-controlled asthma | 202 (63.3%) | 20 (54.1%) | 0.357 |
| Intermediate asthma | 49 (15.4%) | 6 (16.2%) |  |
| Not well-controlled asthma | 68 (21.3%) | 11 (29.7%) |  |

LABA: long-acting beta-agonist.

ACQ assesses the frequency of 5 asthma symptoms during the previous week on a 7-level Likert scale from 0 (no impairment) to 6 (maximum impairment). The overall score, calculated as the mean item responses, ranges from 0 to 6. Cut-off points of 1.5 and 0.75 define not well- and well-controlled asthma, respectively.

Differences in sociodemographic and clinical characteristics at baseline between the whole ARCA sample and the face-to-face assessment subsample were tested using Chi-square or t-test; ***p<* 0.05.

**Supplementary Table 2.** Mokken scaling analysis of the InTeQ: items’ descriptive statistics, inter-item correlations, and Loevinger’s scalability coefficients

|  |  | **Median**  **[IQR]** | **Skewness** | **Inter-Item Correlations**  **(95% CI)** | | | | |  |
| --- | --- | --- | --- | --- | --- | --- | --- | --- | --- |
|  |  |  |  | **Breathe out fully before** | **Close lips tightly** | **Breathe in**  **deeply** | **Hold breath after** | **Breathe out slowly after** |  |
| **InTeQ 5 items (n = 308)** | |  |  |  |  |  |  |  |  |
|  | |  |  |  |  |  |  |  | **Hi (SE)** |
| Breathe out fully before | | 1.0  [0.0 – 4.0] | 0.37 | 1 |  |  |  |  | 0.329 (0.045) |
| Close lips tightly | | 0.0  [0.0 – 3.0] | 0.91 | 0.06  (-0.06 , 0.17) | 1 |  |  |  | 0.190 (0.054) |
| Breathe in deeply | | 0.0  [0.0 – 0.0] | 2.04 | 0.54**  (0.46 , 0.62) | 0.36**  (0.26 , 0.45) | 1 |  |  | 0.532 (0.056) |
| Hold breath after | | 0.0  [0.0 – 3.0] | 0.78 | 0.45**  (0.36 , 0.53) | 0.10  (-0.01 , 0.21) | 0.68**  (0.61 , 0.73) | 1 |  | 0.373 (0.042) |
| Breathe out slowly after | | 2.0  [0.0 – 4.0] | 0.24 | 0.34**  (0.24 , 0.44) | 0.33**  (0.23 , 0.43) | 0.59**  (0.51 , 0.66) | 0.40**  (0.31 , 0.49) | 1 | 0.391 (0.051) |
|  | |  |  |  |  |  |  |  | **Hs (SE)** |
| InTeQ scale | |  |  |  |  |  |  |  | 0.351 (0.037) |
| **InTeQ 4 items (n = 316)** | | | | | | | | | |
|  | |  |  |  |  |  |  |  | **Hi (SE)** |
| Breathe out fully before | | 1.0  [0.0 – 4.0] | 0.37 | 1 |  |  |  |  | 0.416 (0.050) |
| Breathe in deeply | | 0.0  [0.0 – 0.0] | 2.04 | 0.56**  (0.47 , 0.63) |  | 1 |  |  | 0.615 (0.061) |
| Hold breath after | | 0.0  [0.0 – 3.0] | 0.78 | 0.43**  (0.33 , 0.51) |  | 0.68**  (0.62 , 0.74) | 1 |  | 0.491 (0.048) |
| Breathe out slowly after | | 2.0  [0.0 – 4.0] | 0.24 | 0.33**  (0.23 , 0.42) |  | 0.59**  (0.52 , 0.66) | 0.42**  (0.32 , 0.51) | 1 | 0.412 (0.059) |
|  | |  |  |  |  |  |  |  | **Hs (SE)** |
| InTeQ scale | |  |  |  |  |  |  |  | 0.472 (0.044) |

CI: confidence interval; IQR: interquartile range; Hi: homogeneity between each item and the item set; SE: standard error; Hs: homogeneity among all items of the scale. Median [IQR], skewness, and inter-item correlations were estimated from the 5-level Likert scale response options. **Statistically significant differences.

Mokken scaling analysis (MSA) is recommended when items aim to measure ordinal latent variables, items differ in their difficulty [1], and also when latent variables are operationalized with a small number of items [2]. MSA focuses primarily on assessing whether an item set orders respondents accurately on a continuum representing a latent variable, thus fitting a Double Monotonicity Model or a Monotone Homogeneity Model [2] with three assumptions in the latter: unidimensionality, local independence, and monotonicity. Unidimensionality means that respondents who endorse more ‘difficult’ items (or response options with higher values) are more likely to endorse ‘easier’ items. Local independence is understood as items that are associated with each other only via the latent variable they both measure. Monotonicity means that the probability of endorsing an item should not decrease at higher levels of the latent variable. Items that meet these assumptions form a scale, i.e. measure the same underlying latent variable [1,2].

With MSA, we specifically estimated the statistics of central tendency and dispersion of the items [1], inter-item correlations (to verify that all items were positively correlated [1], ideally, between 0.20 and 0.40 [3]), and multivariate outliers (Mahalanobis D^2^ *p*< 0.001). Unidimensionality was tested by examining homogeneity and by performing an automated selection procedure [4] of items dichotomized into “Always” vs the rest, due to their skewed distribution. Loevinger’s homogeneity coefficient (*Hi* and *Hs*) thresholds are: 0.3–0.4 (weak), 0.4–0.5 (medium), and 0.5–1 (good).

**References**

1. Dima AL. Scale validation in applied health research: tutorial for a 6-step R-based psychometrics protocol. Heal Psychol Behav Med. 2018;6:136–61.
2. van Schuur WH. Mokken Scale Analysis: Between the Guttman Scale and Parametric Item Response Theory. Polit Anal. 2003;11:139–63.
3. Piedmont, R.L. Inter-item Correlations. In: Michalos, A.C., editors. Encyclopedia of Quality of Life and Well-Being Research. Dordrecht: Springer; 2014. p. 3303-04.
4. Hemker BT, Sijtsma K, Molenaar IW. Selection of Unidimensional Scales From a Multidimensional Item Bank in the Polytomous Mokken I RT Model: Appl Psychol Meas. 2016;19:337–52.

**Supplementary Table 3.** Agreement between the observers’ assessments

|  |  |  | **Researcher** | | | **% of agreement (95%CI)** | **Kappa (SE)** |
| --- | --- | --- | --- | --- | --- | --- | --- |
|  |  |  | ++ | + | - |  |  |
| **Pediatrician** | Breathe out fully before | ++ | 5 (13.5%) | 0 (0.0%) | 0 (0.0%) | 100.0 (100.0 - 100.0) | 1.000 (0.000) |
|  |  | + | 0 (0.0%) | 2 (5.4%) | 0 (0.0%) |  |  |
|  |  | - | 0 (0.0%) | 0 (0.0%) | 30 (81.1%) |  |  |
|  | Close lips tightly | ++ | 28 (80.0%) | 0 (0.0%) | 0 (0.0%) | 91.4 (82.2 - 100.7) | 0.695 (0.161) |
|  |  | + | 3 (8.6%) | 1 (2.9%) | 0 (0.0%) |  |  |
|  |  | - | 0 (0.0%) | 0 (0.0%) | 3 (8.6%) |  |  |
|  | Breathe in deeply | ++ | 18 (50.0%) | 0 (0.0%) | 0 (0.0%) | 88.9 (78.6 - 99.2) | 0.816 (0.085) |
|  |  | + | 1 (2.8%) | 8 (22.2%) | 0 (0.0%) |  |  |
|  |  | - | 2 (5.6%) | 1 (2.8%) | 6 (16.7%) |  |  |
|  | Hold breath after | ++ | 7 (19.4%) | 0 (0.0%) | 0 (0.0%) | 77.8 (64.2 - 91.4) | 0.642 (0.106) |
|  |  | + | 2 (5.6%) | 5 (13.9%) | 0 (0.0%) |  |  |
|  |  | - | 2 (5.6%) | 4 (11.1%) | 16 (44.4%) |  |  |
|  | Breathe out slowly | ++ | 5 (13.9%) | 1 (2.8%) | 3 (8.3%) | 86.1 (74.8 - 97.4) | 0.679 (0.127) |
|  |  | + | 0 (0.0%) | 2 (5.6%) | 0 (0.0%) |  |  |
|  |  | - | 1 (2.8%) | 0 (0.0%) | 24 (66.7%) |  |  |

++: Correctly performed; +: Poorly performed; -: Not performed; SE: standard error.

Kappa coefficient values were interpreted as 0.0-0.2 (slight agreement), 0.2-0.4 (fair), 0.4-0.6 (moderate), 0.6-0.8 (substantial), and 0.8-1.0 (almost perfect) [1].

**Reference**

1. Landis JR, Koch GG. The measurement of observer agreement for categorical data. Biometrics. 1977;33:159–74.
